# Tingbjerg Changing Diabetes: experiencing and navigating complexity in a community-based health promotion initiative in a disadvantaged neighbourhood in Copenhagen, Denmark

**DOI:** 10.1186/s12889-023-15291-w

**Published:** 2023-02-25

**Authors:** Tina Termansen, Paul Bloch, Mette Kirstine Tørslev, Henrik Vardinghus-Nielsen

**Affiliations:** 1 Department of Health Promotion Research, Steno Diabetes Center Copenhagen, Borgmester Ib Juuls Vej 83, 2730 Herlev, Denmark; 2 Department of Health Sciences and Technology, Aalborg University, Selma Lagerløfs Vej 249, 9260 Gistrup, Denmark

**Keywords:** Complex intervention, Community research, Partnership, Navigating complexity, Disadvantaged neighborhood, Health promotion, Supersetting approach

## Abstract

As a response to the complexity of reducing health inequity there has been a rise in community-based health promotion interventions adhering to the principles of complexity thinking. Such interventions often work with adaptive practice and constitute themselves in complex webs of collaborations between multiple stakeholders. However, few efforts have been made to articulate how complexity can be navigated and addressed by stakeholders in practice. This study explores how partners experience and navigate complexity in the partnership behind Tingbjerg Changing Diabetes (TCD), a community-based intervention addressing health and social development in the disadvantaged neighborhood of Tingbjerg in urban Copenhagen. The study provides important insights on the role of context and how it contributes complexity in community-based health promotion.

The study is based on 18 months of ethnographic fieldwork in the local community including participant observations and 9 in-depth interviews with key partner representatives. Findings show that complexity in TCD can be characterized by unpredictability in actions and outcomes, undefined purpose and direction, and differing organizational logics. Factors that support partners’ navigation in complexity include connectivity, embracing a flexible intervention framework, autonomy, and quick responsiveness. The study showcases the interdependency between the intervention and the context of the disadvantaged neighborhood of Tingbjerg and encourages stakeholders and researchers to embrace the messiness of complexity, and to pay attention to ways through which messiness and unpredictability can be handled.

## Background

Health is deeply ingrained in the social contexts of peoples’ everyday lives and influenced by societal, structural and political factors [1–3]. As a response to the complexity of reducing health inequity, there has been a rise in community-based health promotion interventions emphasizing assets, democracy and empowerment through holistic multi-stakeholder approaches [3–10]. Many community-based interventions adhere to the principles of complexity thinking. Such interventions often constitute themselves within a complex web of collaborations between multiple stakeholders [11] and work adaptively to allow actions and outcomes to emerge gradually as a result of an interplay between stakeholders, intervention and context [11–14].

A central point in complexity thinking is that citizens are not seen as someone to be acted upon, but rather as individuals whose behaviors are affected by their interactions with other individuals [11]. Such a perspective acknowledges that complexity is a product of the many unpredictable interactions and responses between individuals engaging in an intervention, including professional practitioners and beneficiaries [11–17]. Few efforts have been made to articulate how complexity can be navigated by stakeholders or can be addressed in practice [11, 18–20]. However, a multi-site study by Henderson and colleagues addressed these questions in relation to partnership formation in a study on depression care [21]. They found that cultivating a partnership identity, adapting to shifting circumstances, and linking organizational levels to strengthen communication were key strategies for partners to navigate complexity [21]. Although such a study provides important insights into the complexities of partnership formation in complex initiatives, there is still a need to understand more deeply how complex partnership dynamics, contextual factors, and diverse organizational structures are experienced and navigated by practitioners in different settings. Drawing on ethnographic fieldwork as a productive way of addressing the complexity of social life [22, 23] the objective of this ethnographic study is to explore how professional stakeholders experience and navigate complexity in a community-based multi-stakeholder intervention implemented in the disadvantaged neighborhood of Tingbjerg in Copenhagen, Denmark. By focusing on navigational practices, this study provides in-depth knowledge on the complexity of context that may strengthen and improve the development and implementation of complex community health interventions.

## Materials and methods

### Tingbjerg Changing Diabetes – a complex community initiative

The present study was conducted within the framework of a large complex community initiative called Tingbjerg Changing Diabetes (TCD) (www.tingbjergchangingdiabetes.dk). TCD was established in 2015 and targets the local community of Tingbjerg, which is an urban neighborhood in Copenhagen, Denmark, with almost 7,000 residents living in appr. 2,600 apartments. Tingbjerg is considered socially disadvantaged due to poor socioeconomic characteristics such as low employment rates and education and income levels [24, 25]. Tingbjerg is also characterized by an overrepresentation of various chronic diseases, including diabetes, which is 2–3 times higher than the average for the entire municipality of Copenhagen [26, 27]. The purpose of TCD is to promote health and well-being among residents of Tingbjerg and ultimately reduce and prevent type 2 diabetes in the neighborhood.

TCD builds on the Supersetting approach [28], which is an intervention framework that promotes the coordinated engagement of multiple stakeholders in the development and implementation of activities across settings in local communities [28, 29]. In practice, this is done by engaging in long-term partnerships with public, civic, private, and academic organizations, and by being responsive to local suggestions for new activities, projects, and working relationships. The Supersetting approach emphasizes the following five overarching principles for the development and implementation of interventions: 1) *integration*, to ensure that activities are implemented through coordinated action across the boundaries of specific settings, 2) *participation,* to ensure that people are motivated to take ownership of processes of developing and implementing activities, 3) *empowerment,* to ensure that people acquire skills and competencies to express and act on their visions and aspirations, 4) *context-sensitivity*, to ensure that the everyday life challenges of citizens and professionals are acknowledged and considered when developing and implementing activities, and 5) *knowledge*, to ensure that scientific knowledge is produced *from* action and used to *inform* action.

TCD may be characterized as a Complex Adaptive System [30]. In such a framework, challenges involve navigating the goals and opinions of multiple stakeholders, including residents, dealing with a dynamic and emergent approach (where unpredictability is to be expected), negotiating strategies, and being responsive and adaptive [17, 30]. Moreover, complexity goes beyond the intervention as the community itself constitutes complexity, involving shifting political agendas and structural changes. By being characterized as disadvantaged, Tingbjerg is subject to much political attention. Over the years, diverse political initiatives have been imposed upon Tingbjerg in efforts to improve conditions or counteract the development of so-called parallel societies [31]. Most of these initiatives originate from national acts targeting disadvantaged neighborhoods such as the Prevention of Parallel Societies Act, the Policing Zones Act and the Daycare for Children Act [32, 33]. Most recently, the construction of new housing has begun in efforts to change the social mix of residents towards more well-educated and affluent families. This has been done at the expense of recreational areas in the neighborhood, including part of the community hub where the TCD partnership has established a base. Living in a disadvantaged neighborhood thus exposes residents to what has been termed spatial stigma, being the negative labels or discourses on disadvantaged neighborhoods [34]. The stigma pertaining to Tingbjerg as disadvantaged impacts on residents’ responses to interventions and this was something TCD had to navigate when developing activities. 

On this basis, the urban neighborhood of Tingbjerg contributes complexity that must be navigated by professional stakeholders and residents alike.

#### Study design

The study applied a Community Action Research (CAR) design [29, 35] combined with in-depth qualitative ethnographic methods. CAR argues for the active and joint involvement of citizens and professional stakeholders, including researchers, in processes of developing and implementing interventions [29, 35]. On this basis, the first author from Steno Diabetes Center Copenhagen actively contributed to the development and implementation of a community restaurant alongside other stakeholders in TCD. She was present in the community hub two–three days per week from January 2020 to August 2021. The remaining authors participated in regular TCD partnership meetings in which research, implementation and actions were discussed.

The action research approach led to an inevitable duality in the first author’s position as both an insider and outsider, both researching and being part of the field of study [36]. However, with an emergent approach and complex context, the action research approach contributed with continuous learning supporting testing and adjustment of the restaurant [37–39]. 

### The TCD partnership

During the study period from 2020–2021, the TCD partnership consisted of the following three core partner organizations:

- FSB, which is a public housing association that administers housing for residents and manages social development schemes to the benefit of the local community. FSB functions as a gatekeeper to the community by providing access to professional stakeholders, residents, and social networks. Social development schemes have been implemented in Tingbjerg since 2007. They are partly financed through housing rentals and every four years residents vote to decide if the schemes should continue for another term [40].
- Steno Diabetes Center Copenhagen, which is a public diabetes research hospital that provides treatment and care for diabetes patients in the capital region of Denmark. It also conducts place-based health promotion research and has been operating in Tingbjerg since 2015.
- Copenhagen Hospitality College, which is a vocational training college that provides formalized training in cooking, nutrition and waiting skills for the hospitality sector. It has been operating in Tingbjerg since 2018.

FSB and Steno Diabetes Center Copenhagen initiated a collaboration in 2015 when TCD was founded. They thus knew each other and had been collaborating on a few projects when the collaboration surrounding the community restaurant began. Copenhagen Hospitality College was integrated into the partnership in 2018 based on local residents’ interests in food and cooking activities and with a prospect of recruiting young people from Tingbjerg to the training college. The Supersetting approach and its principles had not been negotiated in detail before the establishment of the community restaurant. Representatives from each of these organizations formed a Coordination Group for jointly managing and supporting the various TCD projects and activities on a day-to-day basis. The Coordination Group was directed by a Steering Committee of decision-makers from the three partner organizations. While Steno Diabetes Center Copenhagen and Copenhagen Hospitality College were external organizations to the community, FSB was a local stakeholder with profound local knowledge and networks in the neighborhood. Steno Diabetes Center Copenhagen provided initial funding for the collaboration and facilitated the application of the Supersetting approach. However, unfolding and operationalizing the principles of the Supersetting approach was a joint process with ongoing negotiations between the Coordination Group and the Steering Committee. Implementing the principles in the context of the community restaurant was done by Coordination Group partners working in Tingbjerg. In addition to the core partner organizations subject to this study, the TCD partnership involved a larger network of collaborating organizations from the public, private, civic, and academic sectors.

### Study location

The TCD Coordination Group resided in a former kindergarten in Tingbjerg, which was converted into a community hub for social development and health promotion in the community. The Coordination Group worked with different projects during the time of conducting the present study, although the main activity was a community restaurant in the community hub. The purpose of the restaurant was to involve residents in a gastronomic commune to promote community engagement and action competence. It consisted of cooking workshops and restaurant dining, all prepared by residents under the supervision of a chef from Copenhagen Hospitality College. The restaurant was initiated as a project with no immediate end-date to allow for continuous development and adaptation in response to local needs and interests. At the time of the study, the restaurant was intended to become self-sustaining over time with a gradual takeover by the community The present study was carried out in the community restaurant and the community hub.

### Study participants and data generation

Data was generated from participant observation and semi-structured qualitative interviews with members of TCD Coordination Group consisting of one partner from Copenhagen Hospitality College, one partner from Steno Diabetes Center Copenhagen and two partners from FSB. Other staff members participating ad hoc in the coordination group meetings were also subject to observations. These are all referred to as partners throughout the article. In addition, data was obtained from members of the TCD Steering Group (referred to as leaders) consisting of one decision-maker from each partner organization. Through her active role in the process of establishing the community restaurant, the first author had easy access to all study participants.

The following data was generated:

1. Observation notes from participation in weekly meetings of the Coordination Group as well as occasional meetings between the Coordination Group and research partners.
2. Observation notes from participation in the community restaurant during cooking and dining as well as time spent in the community hub.
3. Seven semi-structured interviews with four partners representing the three TCD partner organizations, namely one project coordinator from Steno Diabetes Center Copenhagen, one chef from Copenhagen Hospitality College, and two social workers from FSB. In addition, two leaders were interviewed, namely one from Copenhagen Hospitality College and one from FSB.
4. Two follow-up focus-group interviews with three partners, one from each partner organization.

Participant observations were carried out to obtain rich contextual data on the setting of Tingbjerg, partner motivations and interactions, collaboration processes and the development of the restaurant. The first author’s participation in the coordination group provided an embodiment of the field, where not only verbal or written first-hand narratives made up the data material, but where a sense of being part of the process strengthened the contextual understanding of complexity pertaining to the establishment of the restaurant [41]. Field notes were taken during and after meetings with partners using an observation guide focusing on partnership dynamics, development process, and barriers and potentials. A separate guide was used for the restaurant evenings to focus specifically on the progress and development of the community restaurant. Observations were supplemented by ethical reflections.

Individual semi-structured interviews were conducted individually with all partners in the Coordination Group and two of the leaders when the restaurant was established in the spring of 2020. These interviews focused on expectations and perspectives on the community restaurant, its concept, scope, and development process. Approximately 9 and 12 months later in the winter and spring of 2021 two focus group interviews were carried out with partners focusing on retrospective reflection on the development of the community restaurant. These interviews also allowed for cross-organizational discussions and reflection [42]. Leaders were not interviewed in this round as they had not been part of the day-to-day process of developing the restaurant. In addition, in the spring of 2021, a final individual interview was carried out with a representative of FSB who had an active role in the restaurant. The inside knowledge of the first author and her familiarity with study participants made interviews as much a process of collective reflection and dialogue as a tool for generating data [43]. Two individual interviews and one focus group took place online due to COVID-19 restrictions while the remaining interviews took place in the community hub or FSB offices in Tingbjerg.

### Data analysis

Interviews were transcribed ad verbatim in NVivo 12. We applied thematic analysis [44] of the data to identify central themes and patterns related to the characteristics of the intervention and how practitioners navigated complexity. The approach was abductive to allow central themes to emerge from initial codes and be explored in more detail over time in an ongoing dialogue with core concepts and theories [45]. In a first round of analysis, which was carried out after the first round of interviews and observations, complexity was applied as an analytical concept to guide an overall exploration of the characterization of the restaurant and its context. In a second round of analysis, which was carried out after all data had been generated, factors were identified that supported partners navigation of complexity. We identified three themes central to how partners experienced complexity. These were 1) Unpredictability in actions and outcomes, 2) Undefined purpose and direction, and 3) Differing organizational logics. In addition, we identified three factors that supported stakeholders’ navigation in complexity. These were 1) Building connectivity, 2) Embracing a flexible framework for action, and 3) Ensuring autonomy and quick responsiveness.

## Findings

### Experiencing complexity

#### Unpredictability in actions and outcomes

Field note excerpt: Over the last month, we have been talking about the numerous people wishing to collaborate with the community hub, including architects, and students. The group has now decided that the architects are a good fit with the plan to build a mobile kitchen in the hub. We have yet again talked about where to build the mobile kitchen, which we plan to use for cooking classes. It has been an ongoing discussion, because the physical renewal project plans to tear down part of the community hub, but the exact plan is still unsure. We have also postponed many talks about the restaurant because the social development scheme is up for renewal. A rumor that rentals will rise has been spreading this year, forcing employees from the social development scheme to invest most of their time in making sure that residents get the right information about the consequences of voting yes and no to the renewal. Another dominating topic of discussion has been how to handle a number of conflicts that have arisen between some residents due to disagreements over how to talk to each other in the hub. This has resulted in some of them not wanting to come here. In addition, there have been cases of vandalism, most likely caused by kids from the neighborhood. This has led to a discussion about children in general being an issue to be addressed, because they often come to the restaurant unaccompanied by parents (Coordination Group meetings in Tingbjerg’s community hub, May and June 2020).

As the excerpt exemplifies, unpredictability was a central feature of the everyday work with the intervention. Unpredictability was not only about the chaotic nature of building the partnership, but also covered the chaos pertaining to the organizational, social, and political context of Tingbjerg with the many potential collaborators or the physical reconstruction scheme in the neighborhood. The leader of FSB elaborated by explaining how the politicized context made it challenging to navigate the setting:

Tingbjerg is in some ways a crazy place. I mean all the demands that came with the ‘parallel society act’, haven’t made it easier to navigate. There is so much political attention directed at this neighborhood. And a lot of financial interests. I mean the whole situation with new residents moving in […]. How do we make this happen without conflict? (leader, FSB)

The leader mentions the process with the physical reconstruction scheme as an example of a situation, which would potentially bring unpredictable responses from residents. Moreover, partners could never fully predict how residents would respond to activities. These conditions also caused indisputable unpredictability in the course of events in the community hub:

You cannot make an agenda a week before a meeting and expect it to hold. In the meantime, 300 new issues will have emerged that influence our direction. […] With the kitchen, the physical renewal plan and all the political stuff, which is out of our hands (partner, Steno Diabetes Center Copenhagen).

There were always new issues to be dealt with in the partnership and there was full agreement that things would not always turn out as expected. A *partner* from FSB explained how the context of Tingbjerg strongly influenced how activities were defined:

...the context we are in is very changeable […] the context is very powerful. It sort of controls us. We do not just implement something and then it turns out the way we expect. The context controls us for many different reasons. […] so, our ideas are constantly challenged because a certain situation requires a certain consideration (partner, FSB public housing association).

This quote is a reference to what the partner described as *the logics of the context*. With this he referred to certain norms and expectations specific to the context of Tingbjerg that sometimes resulted in sudden changes in plans and outcomes. For instance, a so-called ‘come and go’ behavior, where residents would promptly appear and disappear again as soon as they had finished eating in the restaurant, thus making it hard to retain them. Moreover, conflicts sometimes occurred between residents over unexpected issues; for example, what to do with left-over food, how to manage tools and materials in the community hub, or what duties and responsibilities they had. Even small things, such as a participant bringing a dog to the restaurant or the unpleasant smell of a resident who had not been washed for some time required reflections and could potentially result in reconsidering the rules for using the restaurant. This unpredictability was hard to avoid and a condition the partnership had to tackle continuously.

#### Undefined purpose and direction

It was evident from observations and interviews that the lack of concrete purpose and direction for action posed a challenge to partners:

What I have experienced, is that we have lacked a purpose and more concrete projects […]. I simply cannot remember ever having worked with something so diffuse. After a meeting, I have often been in doubt as to whether we agree on the direction (partner, FSB public housing association).

As the quote implies, the partner experienced that the approach made it difficult to agree on a direction and to know exactly how roles and responsibilities were divided. Many of the initial meetings were characterized by a sense of having to ‘find each other’ including repeated discussions about how to develop the restaurant and explore ideas.

The partner from Steno Diabetes Center Copenhagen explained that she experienced a lack of organizational structure at first:

Just figuring out how to meet and what to meet about. […] And when is it partnership-level or project-level or coordination group-level? It is difficult constantly having to balance these things (partner, Steno Diabetes Center Copenhagen).

As needs of residents and available resources could not always be predicted, it could be difficult to even plan what to meet about or who to involve as this partner explains. Although the lack of a defined purpose and direction gave space to responsiveness it also presented a challenge, because partners were required to constantly reconsider direction, making it difficult to do long-term planning and establish clear roles.

#### Differing organizational logics

Both partners and leaders at some point referred to the different logics and motivations of each partner organization as something that made it hard to organize the work, make decisions, and take action. As Copenhagen Hospitality College initially joined the partnership to recruit students from Tingbjerg, its leader was mainly interested in establishing a professional kitchen as a venue for offering cooking classes. However, the key interest of the partner from Copenhagen Hospitality College was to explore the wider potentials of the restaurant, while a partner from the public housing association was mainly interested in building a strong community of residents in the community hub. The partner (professional chef) from Copenhagen Hospitality College explained how he had a hard time understanding the logics of the other partners:

So professionally, cooking is my thing. […] I am not from this world. So, for me there is a whole matrix playing out here […]. Often, letters and numbers are churned out, and to start with I just sat and thought ‘what the hell does that mean?’ (partner, Copenhagen Hospitality College).

The quote exemplifies how the logics of each partner organization was not necessarily clear to the others. A partner from the Steno Diabetes Center Copenhagen similarly explained how she initially was challenged by partnering up with such different organizations and had a hard time integrating the health agenda into their shared talks. She experienced a clash between worlds:

We work in a different way at Steno. In a much more structured way, with meetings planned weeks in advance. It is still hard. Being from such different worlds (partner, Steno Diabetes Center Copenhagen).

From observations it was clear that each partner organization had their own way of operating based on the work they were used to do. The Steno partner later described how it had been hard to find her place and know what her role was because the logics and expectations of the other partners differed so much. The leader from Copenhagen Hospitality College also noted that the different organizational logics presented a challenge in the beginning:

At first everyone simply presented their own project ideas […]. Everyone had their own way into this with ideas related to what they were there for individually. [...]. There have definitely been some challenges with alignment. But just because one person uses very theoretical terms which another person doesn’t understand, it doesn’t mean that they don’t want the same thing (leader, Copenhagen Hospitality College).

As this leader mentions, the different organizational logics were both a matter of having different motivations and different ways of expressing themselves. Working in a cross-organizational initiative thus presented challenges with alignment of expectations and finding common ground due to the differing organizational logics.

### Navigating complexity

#### Establishing connectivity

All partners stressed that it had been crucial to them to feel connected to each other before they could effectively work together. A central mediator for this, they explained, was the shared physical location, the community hub, because it supported the establishment of social ties and made it possible to get to know each other both personally and professionally. The Steno partner explained:

It only makes sense to be physically present and be part of it out there (in Tingbjerg). In the beginning, I also felt like the physical location was missing. There was no place to belong. No place where you feel welcome. […] now this place is as much ours as it is theirs […]. Now they are just a phone call away. (partner, Steno Diabetes Center Copenhagen).

The shared location provided the partner with a sense of belonging and it supported the establishment of connectivity within the partnership and with the wider context of Tingbjerg and legitimized more contact with other stakeholders.

Several partners explained how the complex nature of Tingbjerg, including intense political attention, had caused a sense of ‘project-overload’ among local stakeholders who were tired of philanthropical do-gooders going in and out. This made it necessary to work hard to establish an equal and balanced relationship between partners and to show one’s worth. The Steno partner explained:

It has just been really hard to figure out how to not just be perceived as yet another ‘do-gooder’ coming and asking for something, needing help from the social development scheme. So, from my perspective it has been a lot about building relationships and making it clear that we are here for the long haul. (partner, Steno Diabetes Center Copenhagen).

She highlights that it was important for her to establish herself and Steno Diabetes Center Copenhagen as a partner worth collaborating with, who was there with a relatable purpose and could be trusted by other stakeholders and residents. The physical location played a big part in ensuring common ground and synergy in the initial phases, when roles and concrete activities were rather undefined. This meant that ideas could easily be presented to the other partner representatives and be affirmed or dismissed immediately. As one of the partners from FSB explained: ‘*It is just something else spending your days here in the hub. So many spontaneous things happen’*. This also meant that other stakeholders working in the community hub began to engage in conversations about the restaurant and how it could contribute to ‘their’ activities. The time spent together provided grounds for partners to think and act more in relation to other professionals in Tingbjerg and get closer to residents. In addition, it helped external partners legitimize their engagement and find their place in the partnership.

#### Embracing a flexible framework for action

Interviews and observations point to the importance of embracing the flexibility of the Supersetting approach for practitioners’ ability to navigate complexity. Although the flexibility caused frustration in the beginning, the FSB partner explained that the partnership profited from a flexible framework such as the Supersetting approach because it created new possibilities for action:

The Supersetting approach has made a lot of sense. There were a lot of different stakeholders with different purposes, but when they come together in a not too tight framework, things happen (partner, FSB public housing association).

When partners began to connect more, this FSB partner saw an advantage in the Supersetting approach, because it promoted innovation and a way of collaborating that was not as limited as each partner might be in their own organizations. Another FSB partner elaborated by saying that the Supersetting approach provided them with a suitable, flexible framework that contrasted the usual way of doing things. In his view, flexibility was necessary when navigating the complex context of Tingbjerg:

I think what we are doing here is turning things upside down. We are letting the project control things instead of having to deliver on some fixed criteria [..] which have often been formulated by someone else. […] Instead, you turn it upside down and say: ‘We want to see what happens when we do this’. […] This approach is much more fitting […] (partner, FSB public housing association).

This partner mentioned that the Supersetting approach is more sensible than approaches applied by previous projects in Tingbjerg operating with a pre-defined purpose and activities that he found inflexible, and incapable of meeting residents’ needs or account for unpredictability. Rather, ‘testing and seeing what happens’, was an outspoken way of approaching things within the partnership. Partners would every now and then mention that the flexibility made it easier for them to approach residents and build trust because they were not bound by demands about participation or requirements for documentation. This was something they experienced to be the dominating approaches among other organizations working in Tingbjerg. Consequently, the partnership could easily test different restaurant concepts to assess which was most suitable or to add or remove elements if this could benefit residents or if there was an opportunity to involve other stakeholders. It became a way of doing things that required everyone involved to be prepared to experiment and be open towards new ideas and innovative practice.

#### Ensuring autonomy and quick responsiveness

A final factor supporting partners*’* ability to navigate complexity was autonomy in decision making and quick responsiveness. Local circumstances often necessitated that partners had to deal with sudden incidents or to respond promptly to requests or opportunities. On a general level, this could mean that critical situations and conflicts between residents were prioritized at the expense of other obligations. A partner from the public housing association underlined how autonomy, in the form of the freedom given to him by his leader, was vital to him when responding to residents’ needs:

It requires a certain amount of freedom […] to do as I think is best for them (the residents). […] That space to do what I feel fits is completely there, which means that there is no pressure on me to project onto residents. (partner, FSB public housing association).

Another perception shared by partners was that the trust and autonomy shown to them by their leaders increased their motivation, their decision making and consequently how they approached residents, as the quote underlines. Autonomy meant that the community hub and restaurant were run by the partner representatives with limited interference from leaders. Instead, they regularly talked about how best to respond to residents and develop activities, which improved participation and progress. Partners thus held weekly meetings to discuss challenges and possibilities and how they could be addressed. The Steno partner explained how this was an iterative process:

I think it has been a strength that we have (developed the restaurant) from one time to the next. […] Asking ourselves, “where are we now?” Who has participated the last couple of times and what do they like? […]. It is not far from thought to action. Ideas come and okay “let us do this”. And then 1,000 things occur that makes it difficult to carry through, and that cause things to turn out differently […] (partner, Steno Diabetes Center Copenhagen).

She underlined how the constant changes in the environment required them to switch direction and monitor and reflect on the consequences of their adjustments. This also meant that the restaurant would regularly integrate new elements such as children working as waiters or youth acting as hosts when there was a window of opportunity to do so. The quote above stresses how the partner representatives were very much aware of unpredictability as a condition requiring constant reflection and responsiveness. Overall, partners perceived the ability to respond quickly to the needs of residents as an important factor for dealing with the unpredictability of the context. This was supported by the trust and autonomy granted by their respective leaders.

## Discussion

In this study, we explored complexity in practice by looking at a multi-stakeholder partnership working with health promotion in the disadvantaged neighborhood of Tingbjerg in urban Copenhagen, Denmark. Our methodology provided in-depth ethnographic data on the complexity of context and practices within and around the studied partnership. For instance, we showed how complexity was linked to the unpredictability of the structural context of Tingbjerg and internal dynamics and cultures represented in the cross-organizational partnership. In addition, we explored how complexity was navigated from the perspectives of partners. Our exploration of partners’ navigation in complexity showed that connectivity, the flexible framework as well as autonomy and quick responsiveness functioned as mediating structures, helping partners navigate under complex circumstances. Our findings add new knowledge to the field of complex interventions by showing the messiness of complexity and how a flexible and adaptive intervention strategy could serve as a meaningful response to complexity in a local community context. Accordingly, we have provided a contextual perspective on the nature of complexity in a partnership of stakeholders working with health and social development in a disadvantaged neighborhood. As this article addresses processes of navigating complexity, we have not focused on the implications of navigational practices for evaluation. However, we acknowledge the importance of this discussion and the relevance of considering how approaches such as the Supersetting approach can document progress and effect.

### Complexity within and beyond the intervention

Unpredictability is not confined to deprived neighborhoods. However, as this study shows, the unforeseen is likely to be more prominent and all the more necessary to accommodate in settings where social vulnerability prevails, where trust in authorities is low, and where the involvement of socially vulnerable groups is both a priority and a challenge [46, 47]. When approaching communities such as disadvantaged neighborhoods, contextual factors such as territorial stigmatization and political discourses add to the complexity that must be navigated.

In community interventions, stakeholders must navigate the unpredictability in behaviors and expectations of both residents and professionals. In the present study, we observed that complexity was partly caused by the interdependency between partners and their different organizational logics, which initially made it difficult to take action. As the purpose and direction of the intervention were not predefined, the different organizational logics resulted in the partners sometimes going in different directions and not agreeing on priorities. Through partners’ accounts, our study has provided concrete and contextual examples of complexity in practice and shown that complex adaptive systems such as TCD both add complexity because it is emergent and flexible, and simultaneously make space for navigational practices that allow for adaptation through interaction with context. Other studies have shown similar results [11, 13, 14, 48–51].

### Navigating complexity – organization as key

Organizational structures that give an intervention identity, culture, and function are important, because they support a system’s internally coordinated selectivity and thus the system’s ability to make relevant choices [52, 53]. This is especially important in chaotic contexts with many possible actions, so that boundaries for action are defined and regulated according to what is considered meaningful action, given a specific set of circumstances [54, 55]. In TCD, navigating complexity became a matter of organizing the partnership so that it could continuously test solutions and make choices, which adapted to context and fostered participation of residents. Such an approach is important in contexts where participation may be unstable or where responses and behaviors are unpredictable.

The present study showed that the development of connectivity made it easier for partners to approach each other. Connectivity is about how agents in a system connect and relate to one another and the structures that connects relationships [56–58]. Connectivity between partners was about feeling connected professionally, personally and to the same purpose. Mutual interdependency is crucial to the cultivation of a shared partnership identity, something which is highlighted in the literature on complex adaptive systems stating that the relationships between actors are more important than the actors themselves [56]. Much literature on partnerships highlight relationship building as imperative in cultivating cross-organizational partnerships and in gaining access to communities and hard-to-reach citizens [59–62]. The present study not only supports this understanding, but also underlines the importance of establishing oneself as a trustworthy partner in a partnership consisting of internal and external partners who need to work together. When a partnership is initiated with one partner initially being both an outsider of the local community and a driving force of the project (such as Steno Diabetes Center Copenhagen), power structures and the sense of ownership risk being uneven. This was the case in TCD, where Steno Diabetes Center Copenhagen had to work to establish its position in the local community. However, although professional interests differed at first between the three organizations, we showed that spending time and being physically present in the local community supported a shared identity and common purpose, which ultimately supporting shared ownership. The shared identity and long-term presence of partners in the community also strengthened the local integration of the restaurant and thus prevented it from being perceived as just another quick ‘do-gooder’ project.

Our findings confirm notions within complexity thinking, that unpredictability requires a readiness to adjust and work with many possible solutions and change in actions [11–13, 15, 50, 55]. This is part of the reason why complex interventions adhering to the principles of complexity thinking are often described as *emergent*, meaning that the intervention may change over time in response to unpredictable interactions with the surrounding environments [14]. For TCD partners, the ability to navigate unpredictability was supported by the flexibility of the Supersetting approach. Partners perceived the Supersetting approach to be a framework that embraced complexity because it was not embedded in bureaucratic systems with requirements to documentation or standardization. Research points to the advantages of more loosely structured and flexible approaches because, in contrast to more bureaucratic structures, they make it easier to accommodate the needs and motivations of the local community and to secure the involvement of citizens living there [4]. Essentially, a partnership should reflect ‘the culture of the community and not simply replicate a professional culture, which may make participants uncomfortable’ [21]. Autonomy and quick responsiveness were crucial to demonstrate that the incentives of the intervention could be operationalized into action, something that may improve the chances of gaining citizens’ trust in the motives of the help and ultimately improve the chances of a sustainable intervention [63]. This is a testimony to the importance of not only cultivating horizontal trust between partners, but also vertical trust between leaders and staff members to enable partners to respond to residents’ needs and demands.

The experimental approach adopted by the TCD partnership promoted iterative reflection on actions to navigate complexity. This made it easier to prepare for and respond to the unforeseen. In contrast to traditional intervention designs where most learning happens after completing the intervention, our findings point to the benefits of operating through ongoing iterations and reflexive practice in action rather than solely on action to ensure constant learning and feedback to professional stakeholders and citizens [64]. While reflexive practice is considered highly beneficial and perhaps essential in responding to complex and fluid environments [49, 65, 66], little is mentioned about it in the MRC guidelines, which is a widely applied framework for developing and evaluating complex interventions [67]. A framework such as the Supersetting approach, which allows for much reflexive practice to respond to a shifting environment, may be more suitable when developing a complex intervention that requires navigation in complexity. In TCD, reflexive practice was a foundation in the partnership for maintaining a creative environment, which was always prepared to identify new solutions to pressing community challenges.

## Conclusion

The present study demonstrated that complexity in Tingbjerg Changing Diabetes was characterized by unpredictability of the local context and in actions and outcomes, undefined purpose and direction, and differing organizational logics. Factors that supported partners’ navigation in complexity included connectivity, embracing a flexible intervention framework, and ensuring autonomy and quick responsiveness. The study showcased the interdependency between stakeholders, intervention and context when engaging in a complex community-based intervention in a disadvantaged neighborhood. To ensure meaningful action in the context of a disadvantaged neighborhood, practitioners and researchers must embrace the messiness of complexity and ensure attention to ways through which messiness and unpredictability can be handled. Focusing on ways of ensuring connectivity, applying and embracing a flexible framework and ensuring quick responsiveness through autonomy and trust building will make organizations and practitioners more capable of addressing the unavoidable disruptions and unexpected behaviors, needs or events that arise out of complexity and thus being better capable of accommodating the needs of those targeted by the intervention.

## Data Availability

The datasets used and/or analysed during the current study are available from the corresponding author on reasonable request.
